# Supplementary material for: Chemical Constituents of the Egg Cases of Tenodera angustipennis (Mantidis ootheca) with Intracellular Reactive Oxygen Species Scavenging Activity
Source: Biomolecules. 2021 Apr 10;11(4):556. doi: 10.3390/biom11040556 (PMC8068885; doi:10.3390/biom11040556)
Supplement: Supplementary file 1 [file biomolecules-11-00556-s001.pdf]

Chemical constituents of the egg cases of *Tenodera angustipennis* (Mantidis ootheca) with intracellular reactive oxygen species scavenging activity

Seung Mok Ryu,[1] Hyeon-hwa Nam,[1] Joong Sun Kim,[1] Jun-ho Song,[1] Young Hye Seo,[1] Hyo Seon Kim,[1] A Yeong Lee,[1] Wook Jin Kim,[1] Dongho Lee,[2] Byeong Cheol Moon,[1] and Jun Lee[1,*].

[1]Herbal Medicine Resources Research Center, Korea Institute of Oriental Medicine (KIOM), Naju 58245, Republic of Korea

[2]Department of Plant Biotechnology, College of Life Sciences and Biotechnology, Korea University, Seoul 02841, Republic of Korea

Authors to whom correspondence should be addressed.

[*]Tel: +82-61-338-7129. Fax: +82-61-338-7135. E-mail: junlee@kiom.re.kr (Jun Lee).

Table of contents

Figure S1: Structures of known compounds (**3–15**) 3

Figure S2: Spectroscopic data of known compounds (**3–14**) 4

Figure S3: GC-MS spectrum of compound **15** 7

Figure S4: ^{1}H NMR spectrum of tenoderin A (**1**) (CD_3OD, 500 MHz) 8

Figure S5: ^{13}C NMR spectrum of tenoderin A (**1**) (CD_3OD, 125 MHz) 9

Figure S6: HSQC NMR spectrum of tenoderin A (**1**) (CD_3OD) 10

Figure S7: HMBC NMR spectrum of tenoderin A (**1**) (CD_3OD) 11

Figure S8: COSY NMR spectrum of tenoderin A (**1**) (CD_3OD) 12

Figure S9: HRESIMS spectrum of tenoderin A (**1**) (CD_3OD) 13

Figure S10: ^{1}H NMR spectrum of tenoderin B (**2**) (CD_3OH, 500 MHz) 14

Figure S11: ^{13}C NMR spectrum of tenoderin B (**2**) (CD_3OH, 125 MHz) 15

Figure S12: HSQC NMR spectrum of tenoderin B (**2**) (CD_3OH) 16

Figure S13: HMBC NMR spectrum of tenoderin B (**2**) (CD_3OH) 17

Figure S14: COSY NMR spectrum of tenoderin B (**2**) (CD_3OH) 18

Figure S15: HRESIMS spectrum of tenoderin B (**2**) 19

Figure S16: Stereomicroscope micrographs showing the ootheca morphology of *Tenodera angustipennis*. (A) Dorsal view; (B) Lateral view; (C) Surface pattern on lateral view; Scale bars = 1 cm (A, B). 1 mm (C) 20

Table S1: Screening of the antioxidant activity of extract (100 μg/mL) and isolated compounds (100 μM) 21

Figure S1: Structures of known compounds (**3**–**15**)

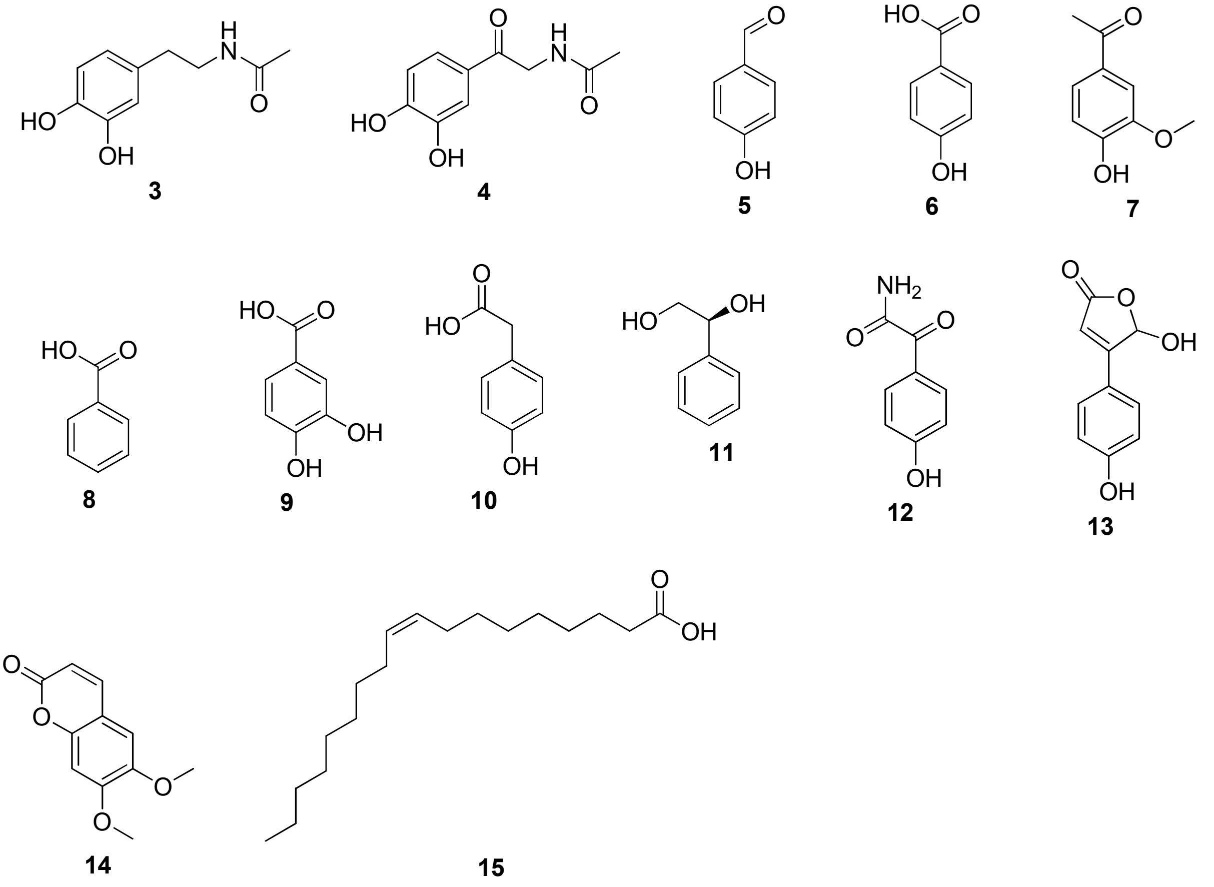

Figure S2: Spectroscopic data of known compounds (**3**–**14**)

N-acetyldopamine (**3**): ESIMS (positive) *m*/*z* 196 $[M + H]^+$; ESIMS (negative) *m*/*z* 194 $[M - H]^-$; ^{1}H NMR (CD_3OD, 500 MHz) δ 6.68 (1H, d, *J* = 8.1 Hz, H-5), 6.64 (1H, d, *J* = 2.1 Hz, H-2), 6.52 (1H, dd, *J* = 8.0, 2.1 Hz, H-6), 3.31 (2H, overlap with MeOD, H-8), 2.62 (2H, t, *J* = 7.4 Hz, H-7), 1.90 (3H, s, H-10); ^{13}C NMR (CD_3OD, 125 MHz) δ 173.4 (C-9), 146.4 (C-3), 144.9 (C-4), 132.2 (C-1), 121.2 (C-6), 117.0 (C-2), 116.5 (C-5), 42.6 (C-8), 36.0 (C-7), 22.7 (C-10).

2-oxo-*N*-acetyldopamine (**4**): ESIMS (positive) *m*/*z* 210 $[M + H]^+$; ESIMS (negative) *m*/*z* 208 $[M - H]^-$; ^{1}H NMR (CD_3OD, 500 MHz) δ 7.45 (1H, d, *J* = 8.7 Hz, H-6), 7.42 (1H, s, H-2), 6.84 (1H, d, *J* = 8.2 Hz, H-5), 4.60 (2H, s, H-8), 2.05 (3H, s, H-10); ^{13}C NMR (CD_3OD, 125 MHz) δ 194.9 (C-7), 173.9 (C-9), 152.8 (C-4), 146.8 (C-3), 128.7 (C-1), 122.9 (C-6), 116.2 (C-5), 115.8 (C-2), 46.9 (C-8), 22.6 (C-10).

4-hydroxybenzaldehyde (**5**): ESIMS (positive) *m*/*z* 123 $[M + H]^+$; ESIMS (negative) *m*/*z* 121 $[M - H]^-$; ^{1}H NMR (CD_3OD, 500 MHz) δ 9.79 (1H, s, H-7), 7.80 (2H, d, *J* = 8.7 Hz, H-2 and H-6), 6.94 (2H, d, *J* = 8.5 Hz, H-3 and H-5); ^{13}C NMR (CD_3OD, 125 MHz) δ 193.0 (C-7), 165.4 (C-4), 133.6 (C-2 and C-6), 130.5 (C-1), 117.0 (C-3 and C-5).

4-hydorxybenzoic acid (**6**): ESIMS (positive) *m*/*z* 139 $[M + H]^+$; ESIMS (negative) *m*/*z* 137 $[M - H]^-$; ^{1}H NMR (CD_3OD, 500 MHz) δ 7.87 (2H, d, *J* = 8.4 Hz, H-2 and H-6), 6.82 (2H, d, *J* = 8.1 Hz, H-3 and H-5); ^{13}C NMR (CD_3OD, 125 MHz) δ 169.8 (C-7), 163.5 (C-4), 133.3 (C-2 and C-6), 123.7 (C-1), 116.2 (C-3 and C-5).

Apocynin (**7**): ESIMS (positive) *m*/*z* 167 $[M + H]^+$; ESIMS (negative) *m*/*z* 165 $[M - H]^-$; ^1H NMR (CD_3OD, 500 MHz) δ 7.56 (1H, dd, J = 8.5, 2.1 Hz, H-6), 7.42 (1H, s, H-2), 7.02 (1H, d, J = 8.4 Hz, H-5), 3.94 (3H, s, OCH_3-3), 2.53 (3H, s, H-8); ^{13}C NMR (CD_3OD, 125 MHz) δ 199.7 (C-7), 153.8 (C-4), 147.7 (C-3), 131.7 (C-1), 123.2 (C-6), 115.7 (C-5), 111.7 (C-2), 56.5 (OCH_3-3), 26.4 (C-8).

Benzoic acid (**8**): ESIMS (negative) *m*/*z* 121 $[M - H]^-$; ^1H NMR (CD_3OD, 500 MHz) δ 8.01 (2H, d, J = 8.0 Hz, H-2 and H-6), 7.57 (1H, t, J = 7.4 Hz, H-4), 7.46 (1H, t, J = 7.5 Hz, H-3 and H-5); ^{13}C NMR (CD_3OD, 125 MHz) δ 170.1 (C-7), 133.9 (C-2 and C-6), 131.2 (C-4), 130.6 (C-1), 129.6 (C-3 and C-5).

Protocatechuic acid (**9**): ESIMS (positive) *m*/*z* 155 $[M + H]^+$; ESIMS (negative) *m*/*z* 153 $[M - H]^-$; ^1H NMR (CD_3OD, 500 MHz) δ 7.42 (2H, overlap with H-2 and H-6), 6.79 (1H, d, J = 7.9 Hz, H-5); ^{13}C NMR (CD_3OD, 125 MHz) δ 170.6 (C-7), 151.6 (C-4), 146.2 (C-3), 124.1 (overlap, C-1 and C-6), 117.9 (C-2), 115.9 (C-5).

4-hydroxyphenylacetic acid (**10**): ESIMS (negative) *m*/*z* 151 $[M - H]^-$; ^1H NMR (CD_3OD, 500 MHz) δ 7.08 (2H, d, J = 8.3 Hz, H-2 and H-6), 6.72 (2H, d, J = 8.5 Hz, H-3 and H-5), 3.50 (2H, s, H-7); ^{13}C NMR (CD_3OD, 125 MHz) δ 176.2 (C-8), 157.6 (C-4), 131.5 (C-2 and C-6), 127.1 (C-1), 116.3 (C-3 and C-5), 41.7 (C-7).

(*S*)-1-phenylenthane-1,2-diol (**11**): $[\alpha]^{26}_D$ +10.8 (*c* 0.01, MeOH); ESIMS (positive) *m*/*z* 139 $[M + H]^+$; ESIMS (negative) *m*/*z* 137 $[M - H]^-$; ^1H NMR (CD_3OD, 500 MHz) δ 7.37 (2H, d, J = 7.1 Hz, H-2 and H-6), 7.33 (2H, t, J = 7.5 Hz, H-3 and H-5), 7.25 (1H, t, J = 7.3 Hz, H-4), 4.68 (1H, t, J = 5.5 Hz, H-7), 3.60 (2H, overlap with MeOH, H-8); ^{13}C NMR (CD_3OD, 125 MHz) δ 143.5 (C-1), 129.4 (C-3 and C-5), 128.7 (C-4), 127.6 (C-6), 76.1 (C-7), 68.9 (C-8).

4-hydroxyphenylglyoxylic acid amide (**12**): ESIMS (negative) *m*/*z* 164 [M – H]$^-$; ^{1}H NMR (CD_3OD, 500 MHz) δ 8.01 (2H, d, *J* = 8.8 Hz, H-2 and H-6), 6.87 (2H, d, J = 8.9 Hz, H-3 and H-5); ^{13}C NMR (CD_3OD, 125 MHz) δ 189.8 (C-7), 170.2 (C-8), 165.4 (C-3), 134.3 (C-2 and C-6), 126.1 (C-1), 116.7 (C-3 and C-5).

(±)-hydroxybutenolide (**13**): Racemic mixture, ESIMS (positive) *m*/*z* 193 [M + H]$^+$; ESIMS (negative) *m*/*z* 191 [M – H]$^-$; ^{1}H NMR (CD_3OD, 500 MHz) δ 7.67 (2H, d, *J* = 8.8 Hz, H-2 and H-6), 6.86 (2H, d, *J* = 8.8 Hz, H-3 and H-5), 6.50 (1H, s, H-2′), 6.32 (1H, s, H-4′); ^{13}C NMR (CD_3OD, 125 MHz) δ 174.3 (C-5′), 165.8 (C-3′), 162.3 (C-4), 131.5 (C-2 and C-6), 122.4 (C-1), 117.0 (C-3 and C-5), 111.9 (C-4′), 100.1 (C-2′).

Scoparone (**14**): ESIMS (positive) *m*/*z* 207 [M + H]$^+$; ^{1}H NMR (CD_3OD, 500 MHz) δ 7.90 (1H, d, *J* = 9.4 Hz, H-4), 7.15 (1H, s, H-5), 7.00 (1H, s, H-8), 6.27 (1H, d, *J* = 9.4 Hz, H-3), 3.93 (3H, s, OCH_3-7), 3.88 (3H, s, OCH_3-6); ^{13}C NMR (CD_3OD, 125 MHz) δ 164.0 (C-2), 155.0 (C-7), 151.5 (C-8a), 148.3 (C-6), 146.1 (C-4), 113.7 (C-3), 113.3 (C-4a), 110.1 (C-5), 101.2 (C-8), 57.0 (OCH_3-7), 57.0 (OCH_3-6).

Figure S3: GC-MS spectrum of compound **15**

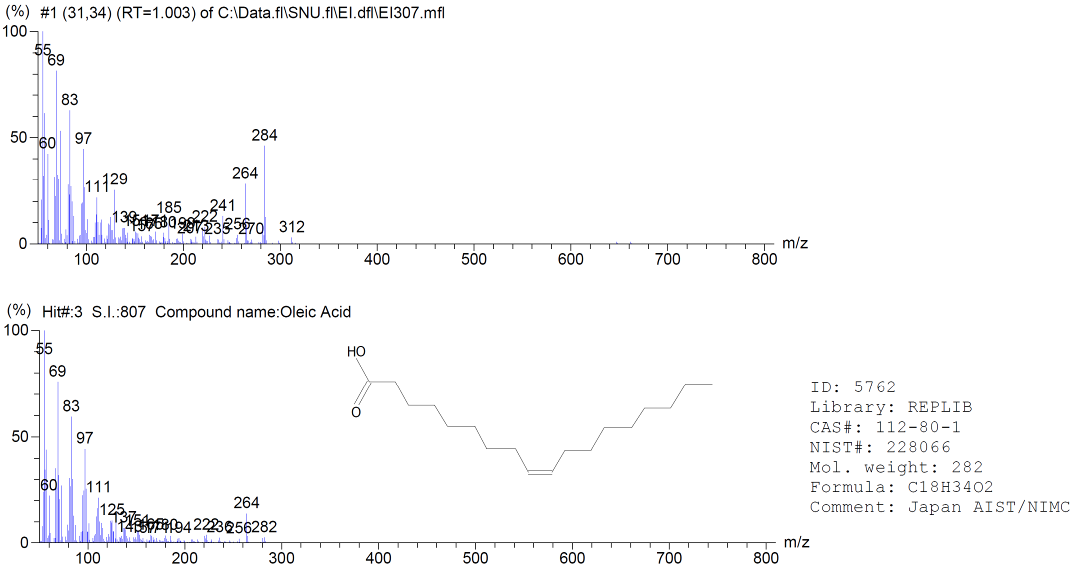

Figure S4: ^{1}H NMR spectrum of tenoderin A (**1**) (CD_3OD, 500 MHz)

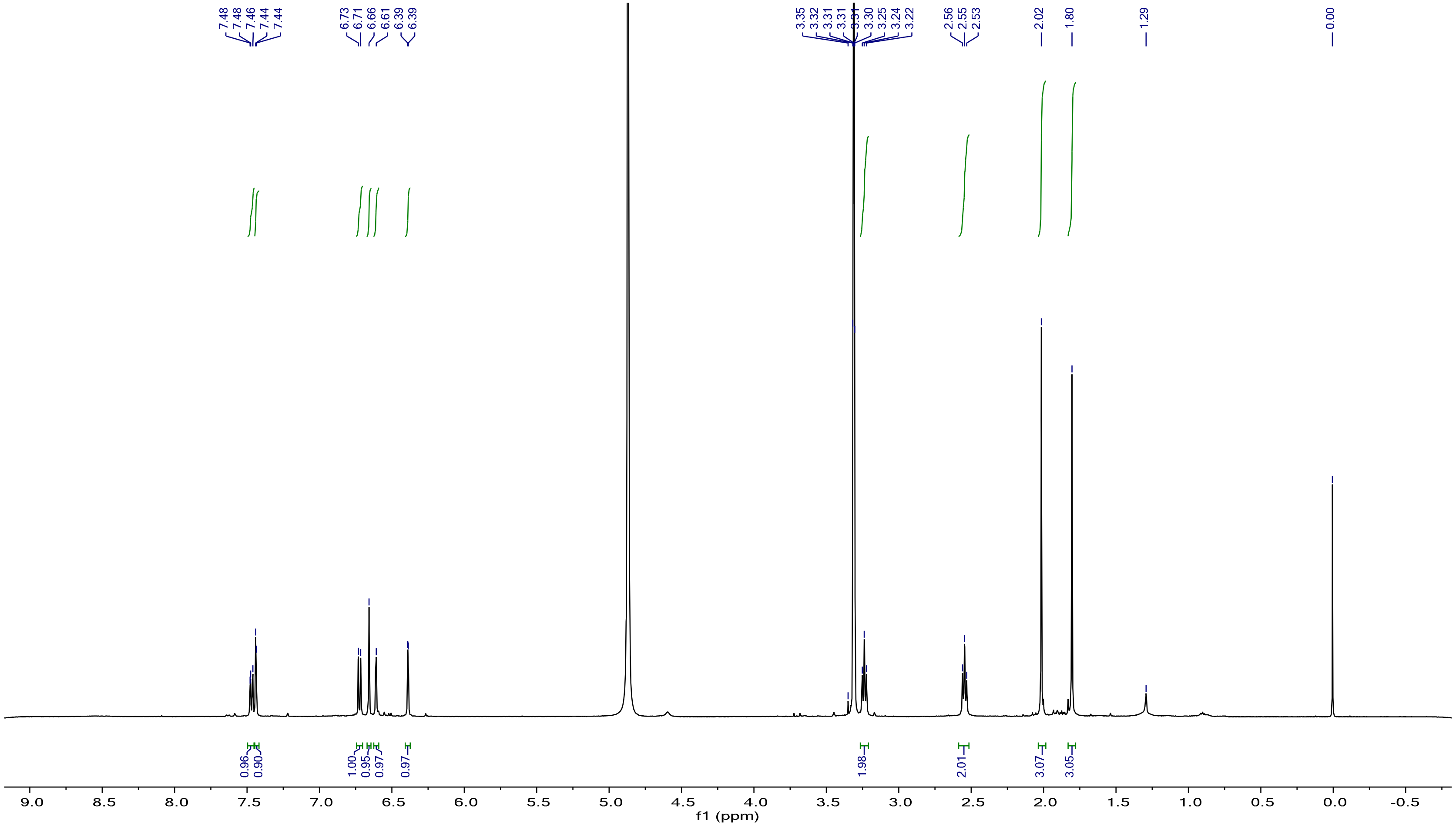

Figure S5: ^{13}C NMR spectrum of tenoderin A (**1**) (CD_3OD, 125 MHz)

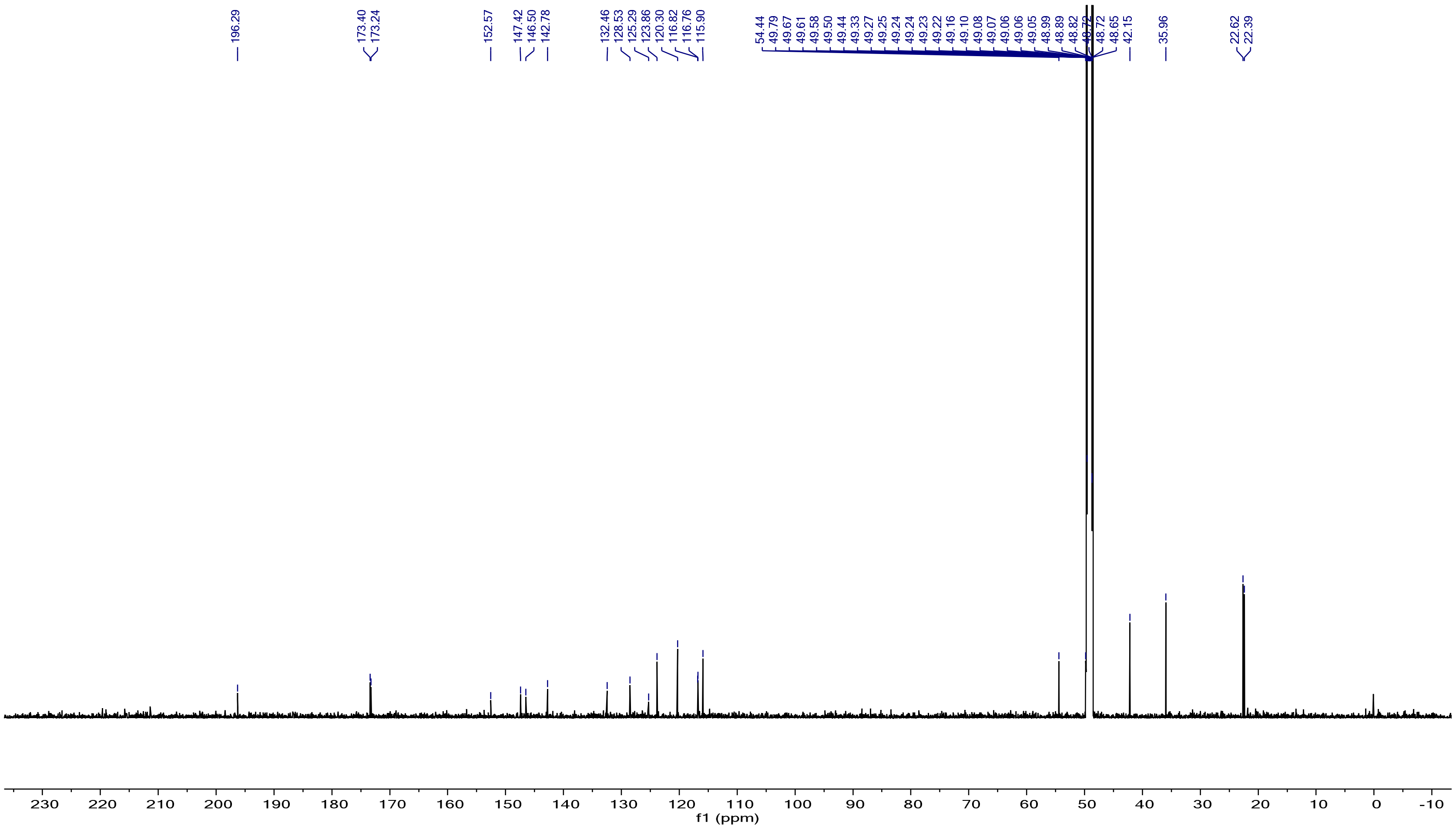

Figure S6: HSQC NMR spectrum of tenoderin A (**1**) (CD_3OD)

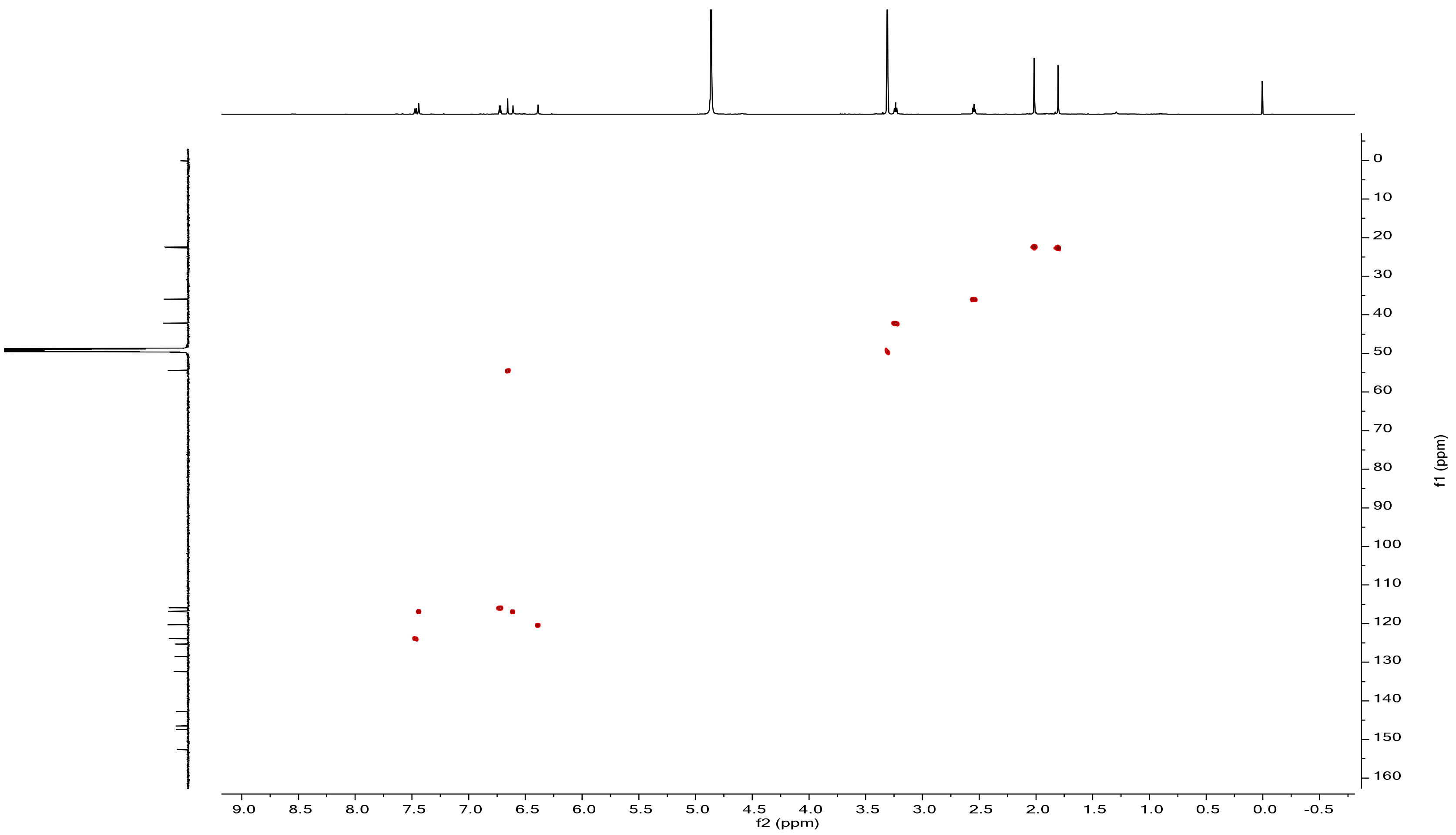

Figure S7: HMBC NMR spectrum of tenoderin A (**1**) (CD_3OD)

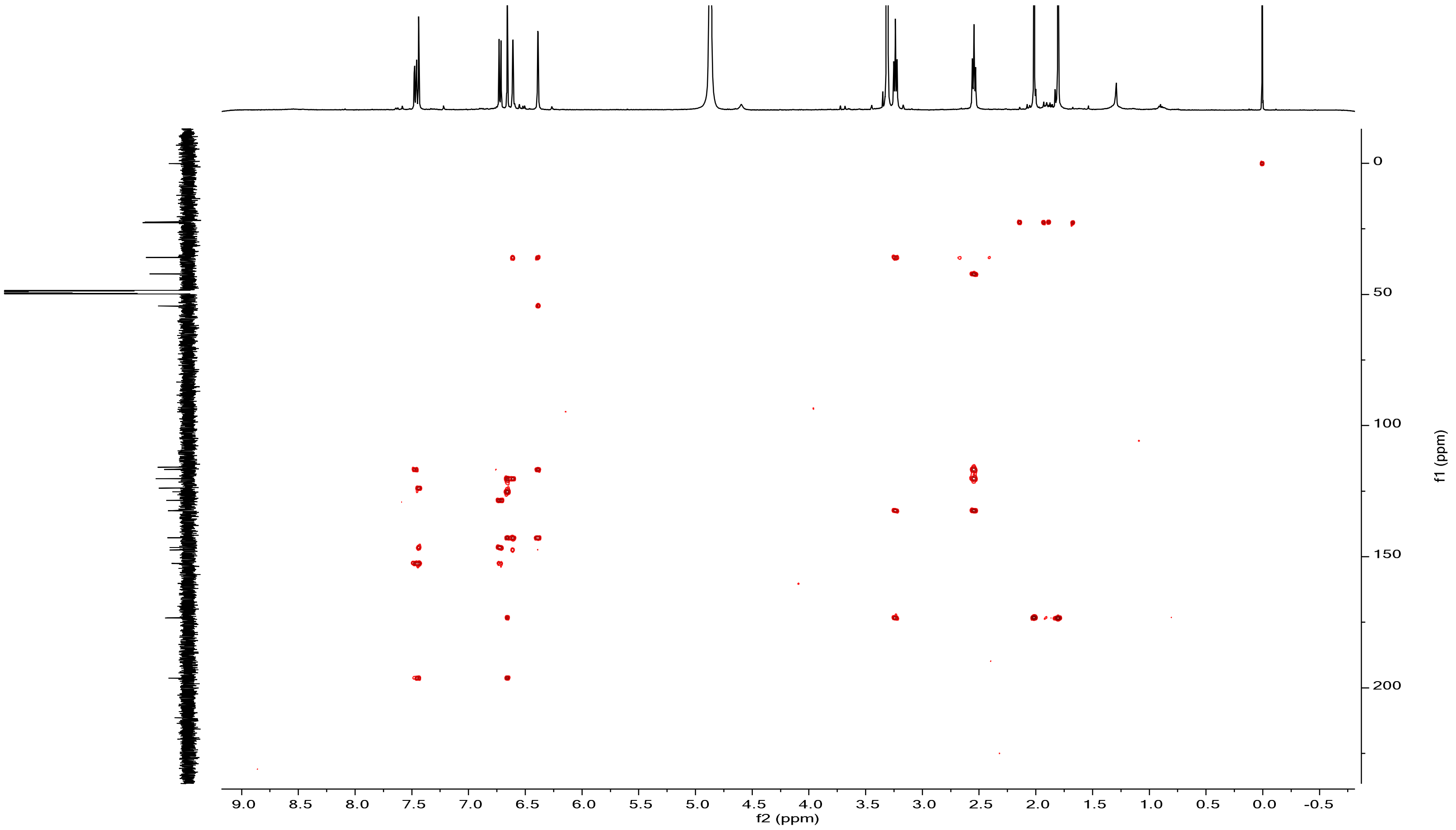

Figure S8: COSY NMR spectrum of tenoderin A (**1**) (CD_3OD)

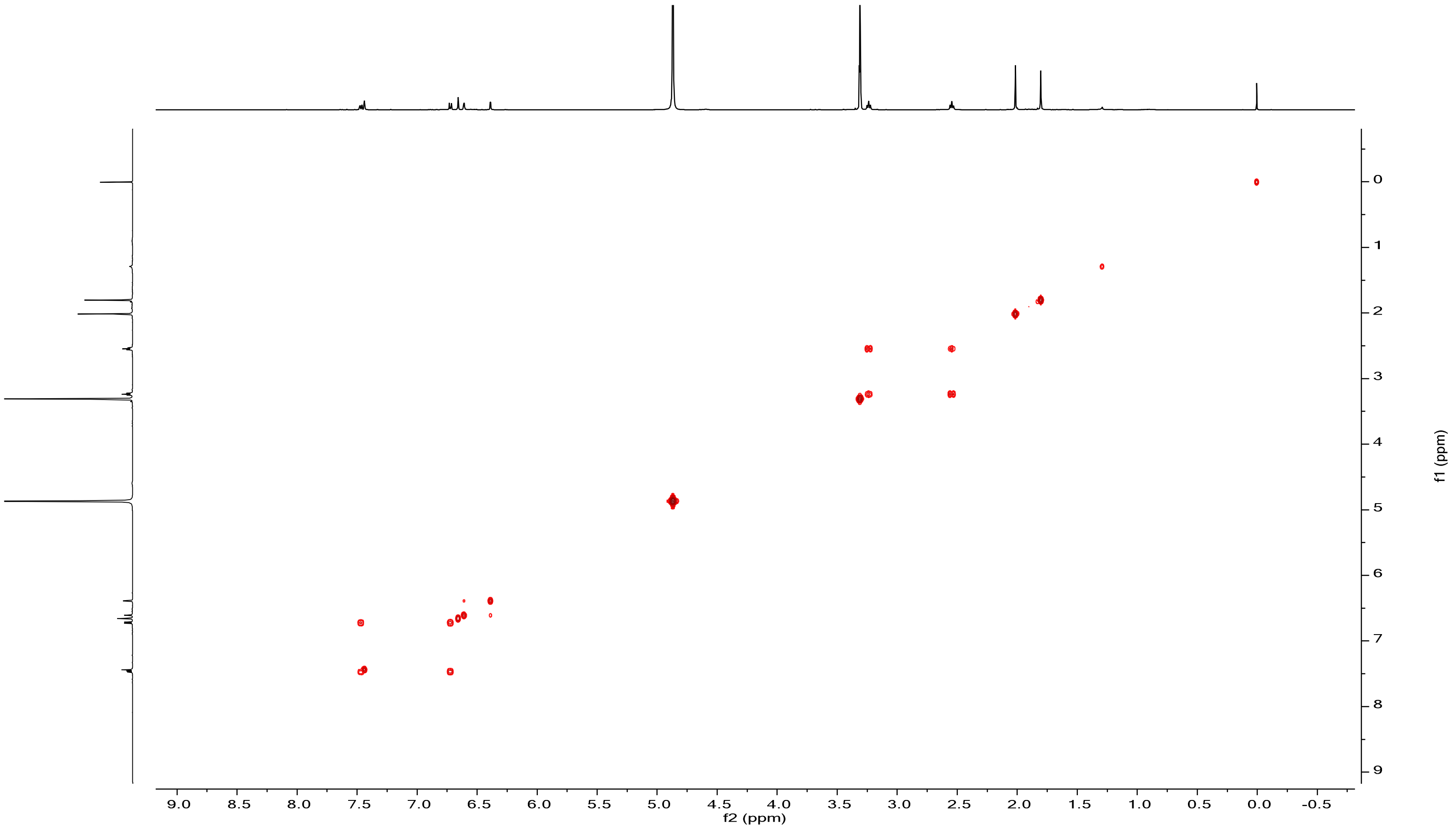

Figure S9: HRESIMS spectrum of tenoderin A (**1**) (CD_3OD)

Elemental Composition Report

Page 1

Tolerance = 5.0 PPM / DBE: min = -1.5, max = 50.0
Element prediction: Off
Number of isotope peaks used for i-FIT = 3

Monoisotopic Mass, Even Electron Ions
1273 formula(e) evaluated with 9 results within limits (up to 50 best isotopic matches for each mass)
Elements Used:
C: 0-500 H: 0-1000 N: 0-200 O: 0-200
200805_SeoYH_SM-56-1K_Neg (0.025) Is (1.00,1.00) C20H22N2O7
1: TOF MS ES-

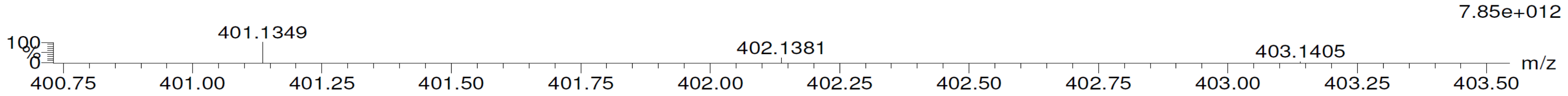

Minimum:	80.00				-1.5				
Maximum:	100.00		5.0	5.0	50.0				
Mass	RA	Calc. Mass	mDa	PPM	DBE	i-FIT	Norm	Conf(%)	Formula
401.1349	100.00	401.1349	0.0	0.0	11.5	46.9	0.000	100.00	C20 H21 N2 O7
		401.1362	-1.3	-3.2	16.5	70.9	23.914	0.00	C21 H17 N6 O3
		401.1335	1.4	3.5	17.5	75.1	28.125	0.00	C17 H13 N12 O
		401.1330	1.9	4.7	24.5	79.1	32.116	0.00	C32 H17
		401.1367	-1.8	-4.5	-1.5	79.4	32.480	0.00	C8 H25 N4 O14
		401.1354	-0.5	-1.2	4.5	82.4	35.444	0.00	C5 H17 N14 O8
		401.1367	-1.8	-4.5	9.5	82.6	35.612	0.00	C6 H13 N18 O4
		401.1340	0.9	2.2	-0.5	82.8	35.902	0.00	C4 H21 N10 O12
		401.1340	0.9	2.2	10.5	86.1	39.154	0.00	C2 H9 N24 O2

Figure S10: ^{1}H NMR spectrum of tenoderin B (**2**) (CD_3OH, 500 MHz)

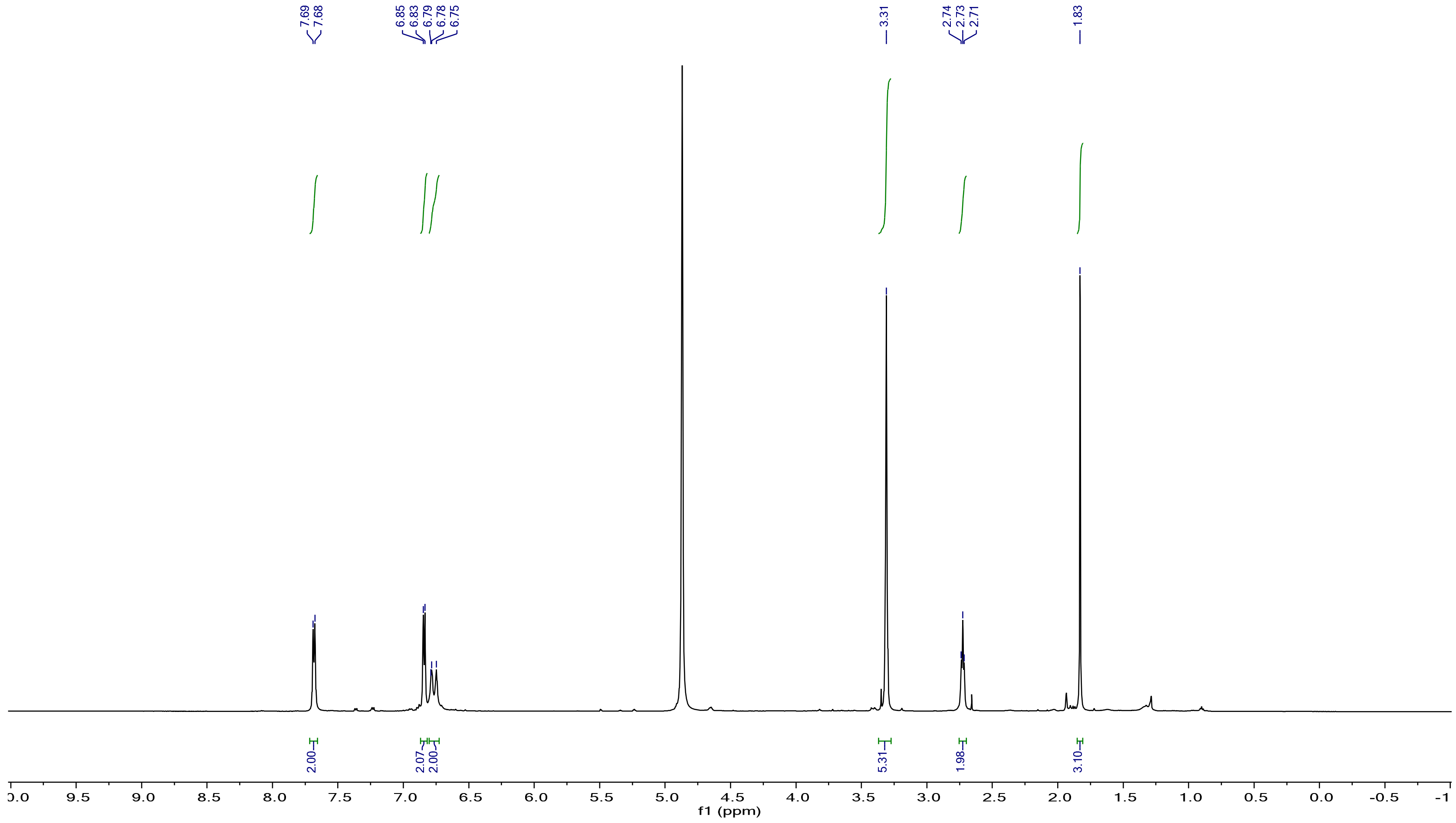

Figure S11: ^{13}C NMR spectrum of tenoderin B (**2**) (CD_3OH, 125 MHz)

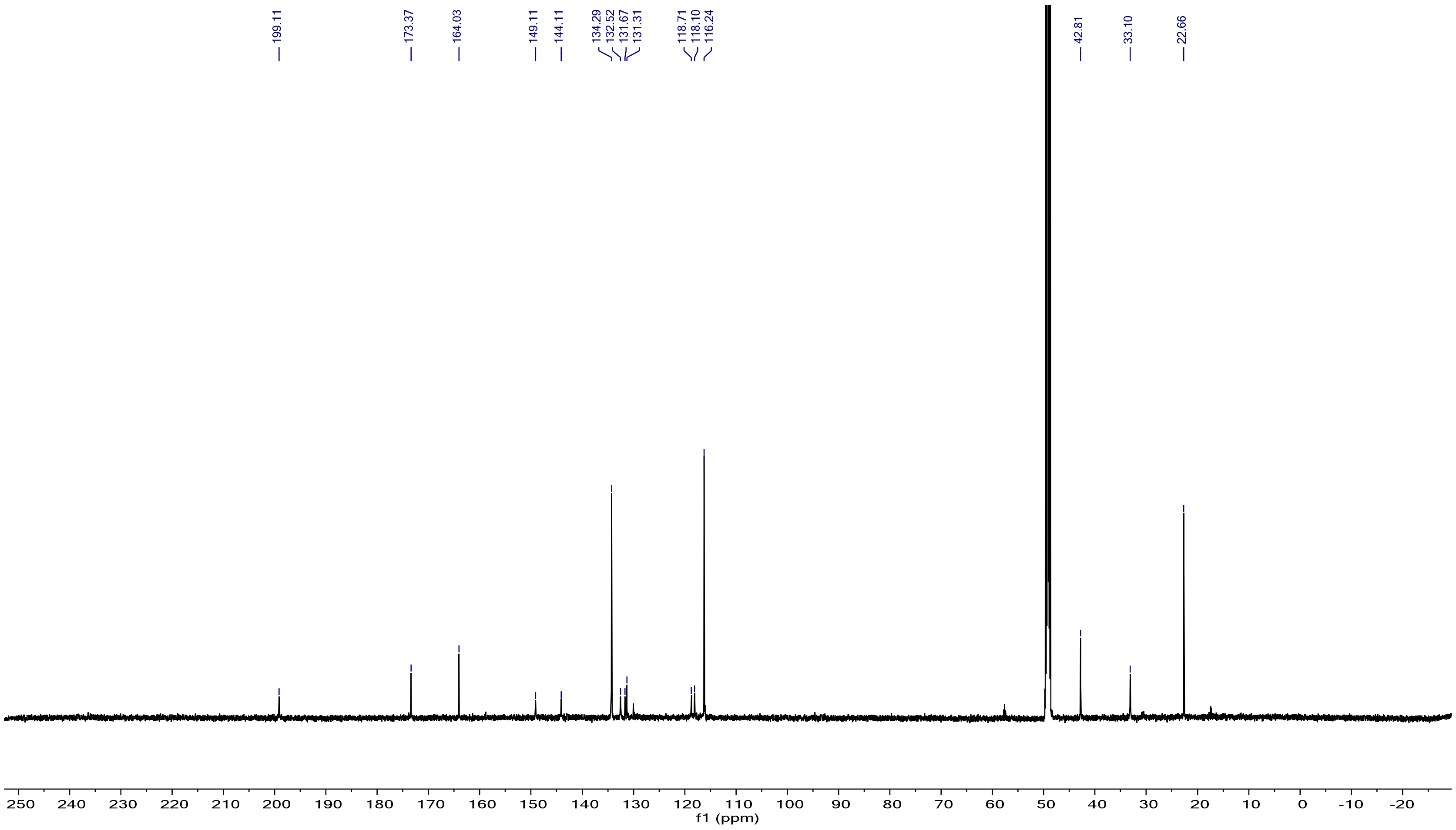

Figure S12: HSQC NMR spectrum of tenoderin B (**2**) (CD_3OH)

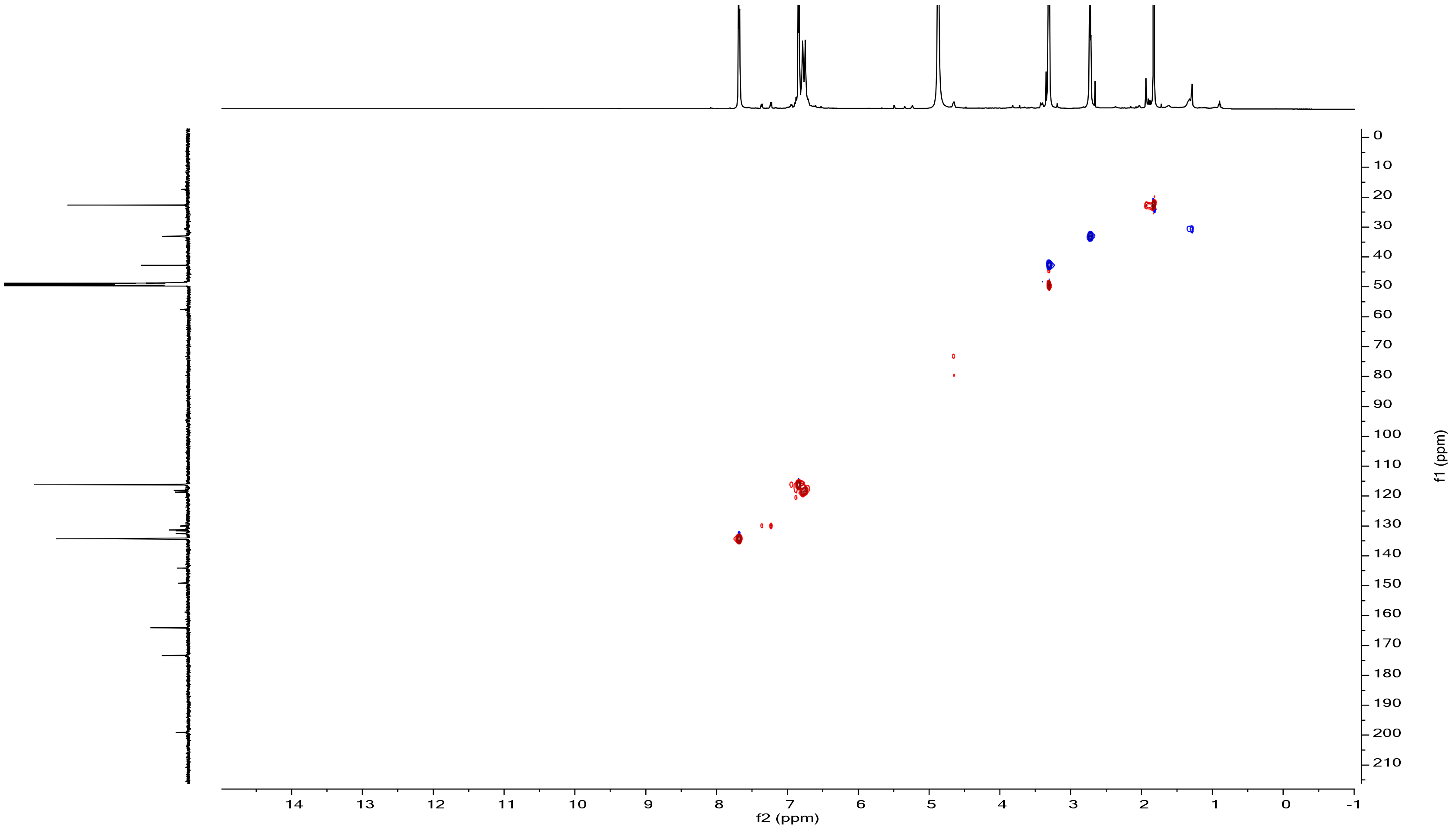

Figure S13: HMBC NMR spectrum of tenoderin B (**2**) (CD_3OH)

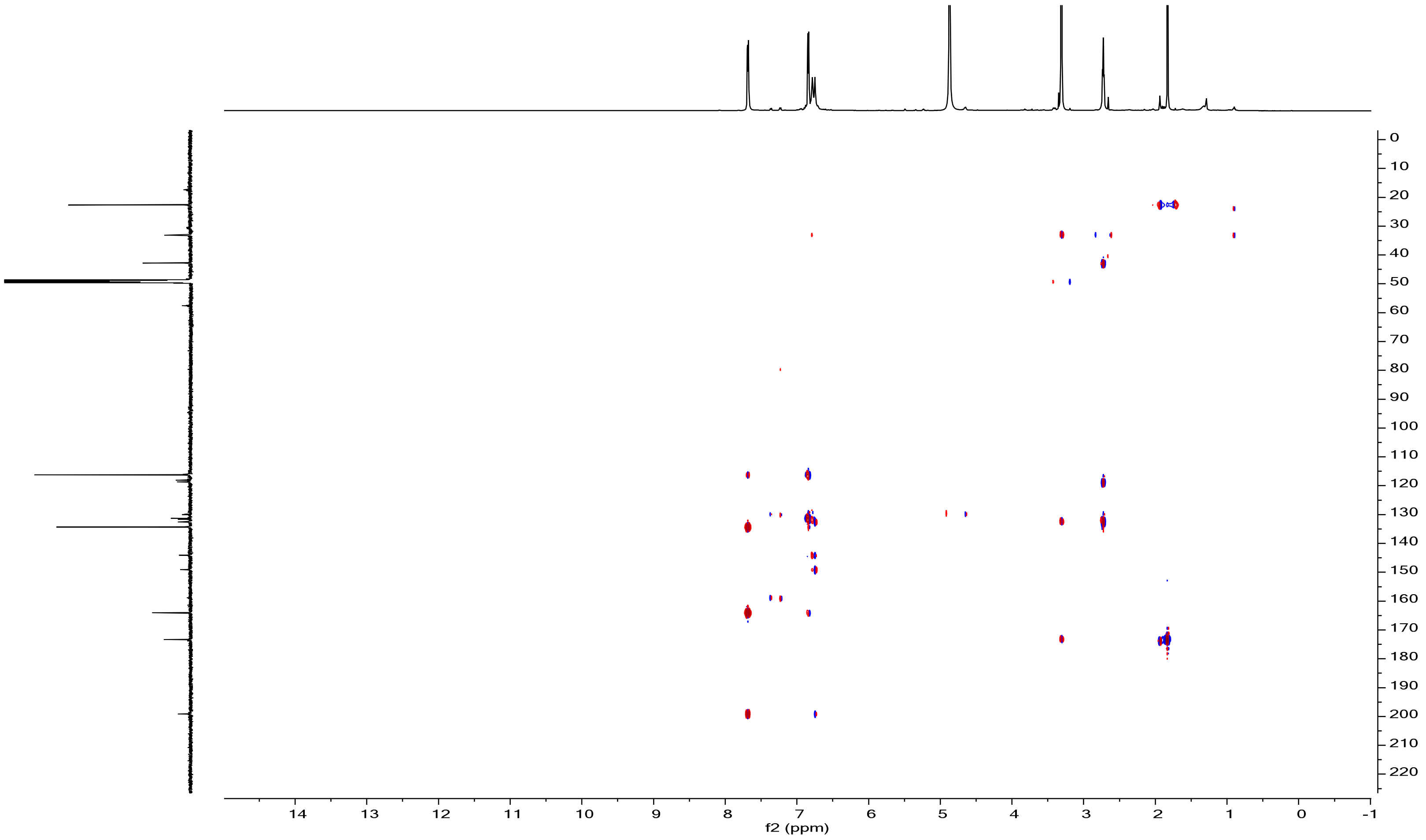

Figure S14: COSY NMR spectrum of tenoderin B (**2**) (CD_3OH)

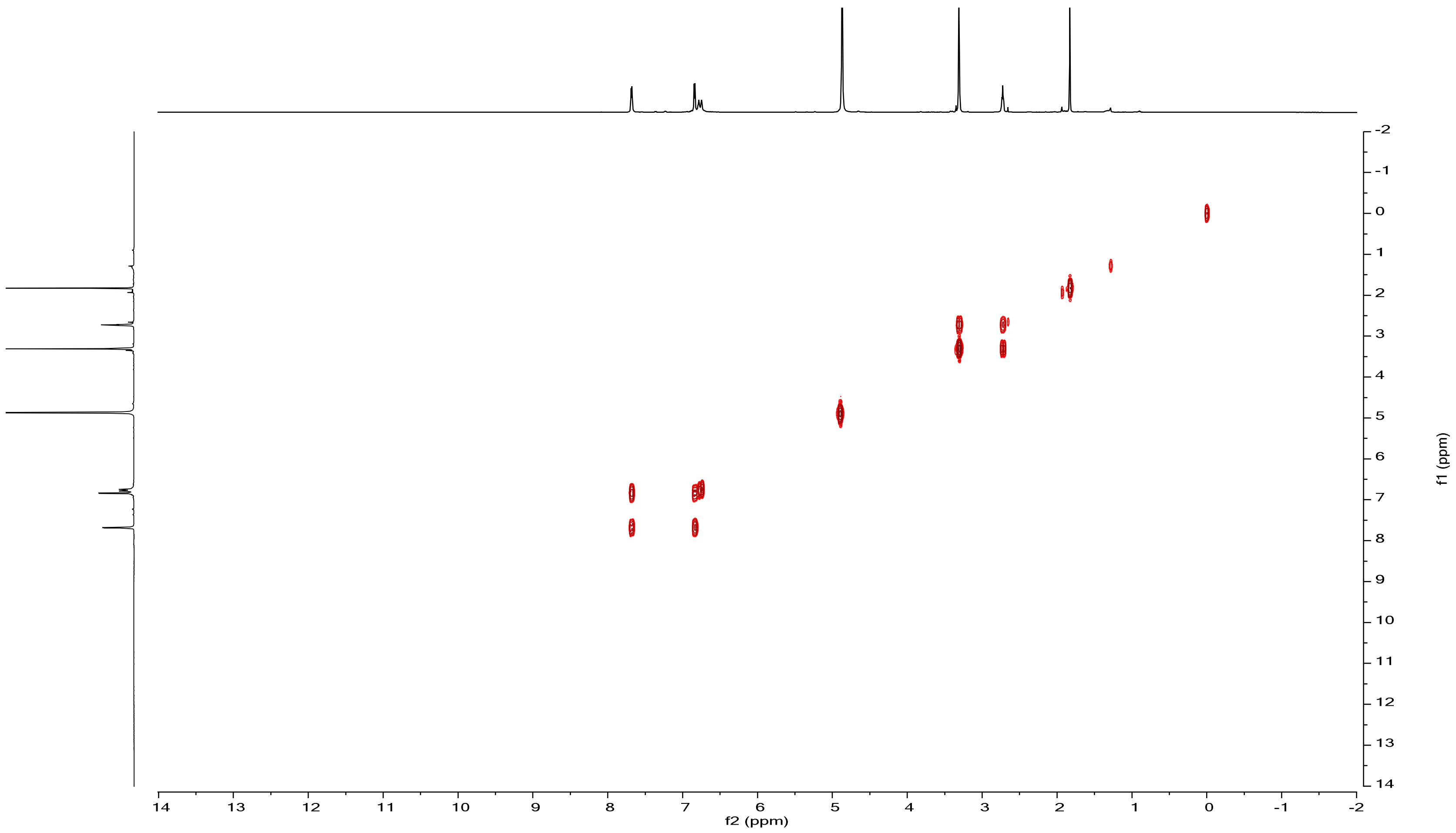

Figure S15: HRESIMS spectrum of tenoderin B (**2**)

Elemental Composition Report

Page 1

Multiple Mass Analysis: 2 mass(es) processed

Tolerance = 5.0 PPM / DBE: min = -1.5, max = 50.0
Element prediction: Off
Number of isotope peaks used for i-FIT = 3

Monoisotopic Mass, Even Electron Ions
659 formula(e) evaluated with 4 results within limits (up to 50 best isotopic matches for each mass)
Elements Used:
C: 0-500 H: 0-1000 N: 0-200 O: 0-200
202805_SeoYH_SM-36-1K_Neg-re 326 (3.701) Cm (320:331)
1: TOF MS ES-

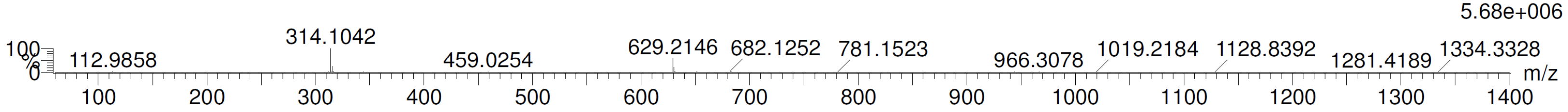

Minimum:	80.00				-1.5				
Maximum:	100.00		5.0	5.0	50.0				

Mass	RA	Calc. Mass	mDa	PPM	DBE	i-FIT	Norm	Conf(%)	Formula
314.1042	100.00	314.1042	0.0	0.0	15.5	1134.2	0.060	94.20	C18 H12 N5 O
		314.1028	1.4	4.5	10.5	1137.0	2.848	5.79	C17 H16 N O5
		314.1047	-0.5	-1.6	8.5	1143.9	9.758	0.01	C3 H8 N17 O2
		314.1034	0.8	2.5	3.5	1144.3	10.142	0.00	C2 H12 N13 O6

Figure S16: Stereomicroscope micrographs showing the ootheca morphology of *Tenodera angustipennis*. (A) Dorsal view; (B) Lateral view; (C) Surface pattern on lateral view; Scale bars = 1 cm (A, B). 1 mm (C)

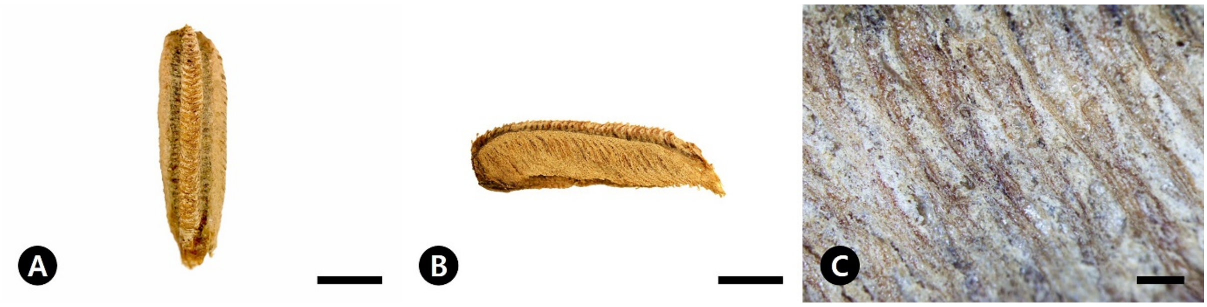

Table S1: Screening of the antioxidant activity of extract (100 μg/mL) and isolated compounds (100 μM)

Compounds	Antioxidant capacity (%)	
	DPPH	ABTS
Extract	81.99 ± 1.98 [1)]	99.74 ± 0.13
**1a**	56.59 ± 0.60	60.56 ± 0.18
**1b**	71.16 ± 0.22	67.77 ± 1.29
**2**	80.83 ± 0.09	93.13 ± 0.31
**3**	76.39 ± 2.60	95.87 ± 0.12
**4**	81.93 ± 0.25	89.53 ± 0.17
**5**	-1.83 ± 0.75	0.93 ± 1.46
**6**	0.37 ± 0.28	-0.12 ± 0.99
**7**	9.40 ± 0.97	1.59 ± 1.23
**8**	-1.00 ± 0.52	1.39 ± 1.27
**9**	78.87 ± 0.48	92.38 ± 0.04
**10**	38.80 ± 1.09	11.45 ± 0.86
**11**	41.58 ± 0.90	19.05 ± 0.48
**12**	-0.53 ± 1.40	2.06 ± 0.69
**13**	-0.03 ± 1.58	0.37 ± 0.12
**14**	-1.74 ± 1.70	0.37 ± 0.26
**15**	-2.40 ± 1.42	-1.79 ± 0.45
Gallic acid	82.89 ± 0.09	95.40 ± 0.07

1) Values are reported as mean ± SD (n=3)
